# How to Argue

**Published:** 1878-02

**Authors:** 


### HOW TO ARGUE.

IT is *prima facie* evidence of a low intelligence or a low nature, or both, in the man who resorts to personalities in controversy; and if I retaliate in kind, I but sink myself to his level, to rise from it no more.

Conscious power is calm. An admirable illustration of this was given by the late Mr. Henderson, a Scotchman, and an eminent Oxford scholar. A collegian, proud of his logical abilities, was anxious to meet Mr. Henderson, but shortly losing his self-possession, he dashed a glass of wine in the Scotchman's face. The latter, coolly wiping it away, replied: "*Sir, this is a digression, now for the argument! !*"

There is perhaps the shadow of an apology for letting drop an epithet or a sarcasm in an oral dispute, but when persons use these, with all the advantage of writing out a controversy, when they become deliberately personal, in the quiet of their own library, we instinctively pity them, as inheriting a rude nature which time and opportunity are powerless to polish.

